# Mucin‐producing urothelial‐type adenocarcinoma of the prostate with sarcomatoid features and novel molecular phenotype

**DOI:** 10.1002/iju5.12672

**Published:** 2023-11-20

**Authors:** Pranav S Renavikar, Thomas J Auen, David G Wagner, Subodh M Lele

**Affiliations:** ^1^ Department of Pathology and Microbiology University of Nebraska Medical Center Omaha Nebraska USA

**Keywords:** mucin‐producing, mutations, prostate, PSA, urothelial

## Abstract

**Introduction:**

Mucin‐producing urothelial‐type adenocarcinoma of the prostate is a rare tumor that may not elevate serum prostate‐specific antigen, creating significant diagnostic and monitoring challenges. We evaluate our case in detail and review prior studies to demonstrate that the pathologic and molecular features of this tumor are distinct from conventional prostate adenocarcinoma.

**Case presentation:**

Our patient had a remote history of radiation‐treated conventional prostate adenocarcinoma and presented many years later with an abscess‐like prostate mass leading to urinary obstruction and hematuria. Biopsy revealed mucin‐producing urothelial‐type adenocarcinoma of the prostate with concurrent sarcomatoid features. Molecular studies showed a unique phenotype involving alterations in the *KRAS*, *PTEN*, *RAD21*, and *TP53* genes.

**Conclusions:**

To our knowledge, this is the first report that describes sarcomatoid features and molecular mutations in mucin‐producing urothelial‐type adenocarcinoma of the prostate.

**Abbreviations & Acronyms**
ADT: androgen deprivation therapy
CEA: carcinoembryonic antigen
CK7: cytokeratin 7
CT: computed tomography
PET: positron emission tomography
PSA: prostate‐specific antigen
TUR: transurethral resection

**Keynote message**

Compared to conventional prostate adenocarcinoma which can be surveilled by serum PSA, mucin‐producing urothelial‐type adenocarcinoma is challenging to diagnose and monitor due to lack of PSA expression, distinct site of origin, and clinical mimics. Urologists and pathologists should keep this unique entity in mind when working up mucinous tumors around the prostatic urethra.

## Introduction

Mucin‐producing urothelial‐type adenocarcinoma of the prostate is a very rare entity that typically develops in the prostatic urethra or proximal large ducts of the prostate.^1^ Appropriate workup of these tumors is important on limited biopsy samples, mainly because they do not express PSA and have significant morphologic and immunohistochemical overlap with the more commonly seen entities like conventional prostate adenocarcinoma with mucin production and secondary adenocarcinoma from urinary bladder or colorectal origin.

Our patient had a remote history of radiation‐treated conventional prostate adenocarcinoma and presented years later with obstructive urinary symptoms and hematuria due to an abscess‐like prostate mass. Upon clinical suspicion of recurrent prostate adenocarcinoma, the mass was biopsied and revealed mucin‐producing urothelial‐type adenocarcinoma of the prostate showing infiltrative atypical glandular cells containing intracytoplasmic mucin, sarcomatoid areas of tumor cells, negative PSA and NKX3.1, and alterations in the *KRAS*, *PTEN*, *RAD21*, and *TP53* genes.

## Case presentation

### Clinical summary

**Fig. 1:**
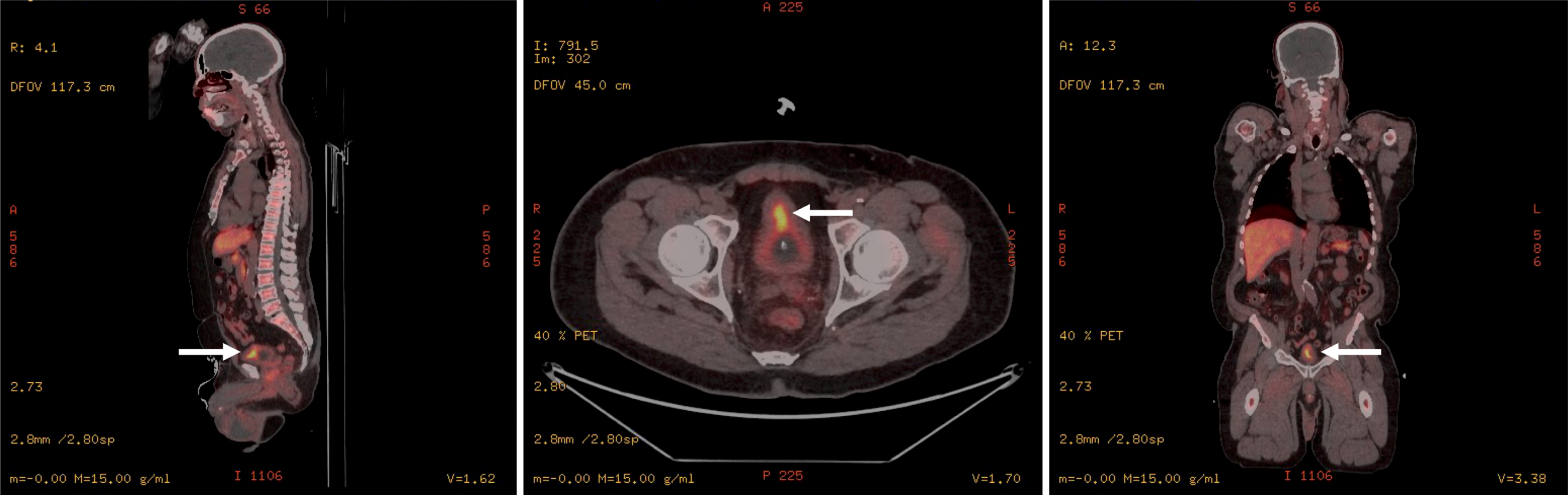
Radiological assessment of the prostate gland mass. PET scans in sagittal, axial, and coronal (left to right) planes demonstrate radiotracer uptake in the prostate gland.

An older male with a history of radiation‐treated prostate adenocarcinoma (Gleason score 3 + 3 = 6, serum PSA of 7.5 ng/mL) 20 years ago and surveilled with annual serum PSA levels presented with hematuria and obstructive urinary retention which required catheterization. Imaging studies showed an abscess‐like mass involving the prostate gland bilaterally and rapidly rising PSA (4.39 ng/mL), suspicious for locally/regionally recurrent prostate cancer (Fig. 1). The colon and urinary bladder showed no evidence of masses.

Following androgen deprivation therapy (ADT), he had an undetectable serum PSA, but his pelvic/urethral pain and discomfort had worsened. Given the lack of clinical improvement after a favorable PSA response, a restaging CT scan of the abdomen and pelvis demonstrated an increased size of the prostate mass. Overall, the findings were worrisome for a non‐conventional malignancy—the differential diagnoses included small cell carcinoma, sarcoma, and secondary metastasis to the prostate gland.

### Pathologic evaluation

**Fig. 2:**
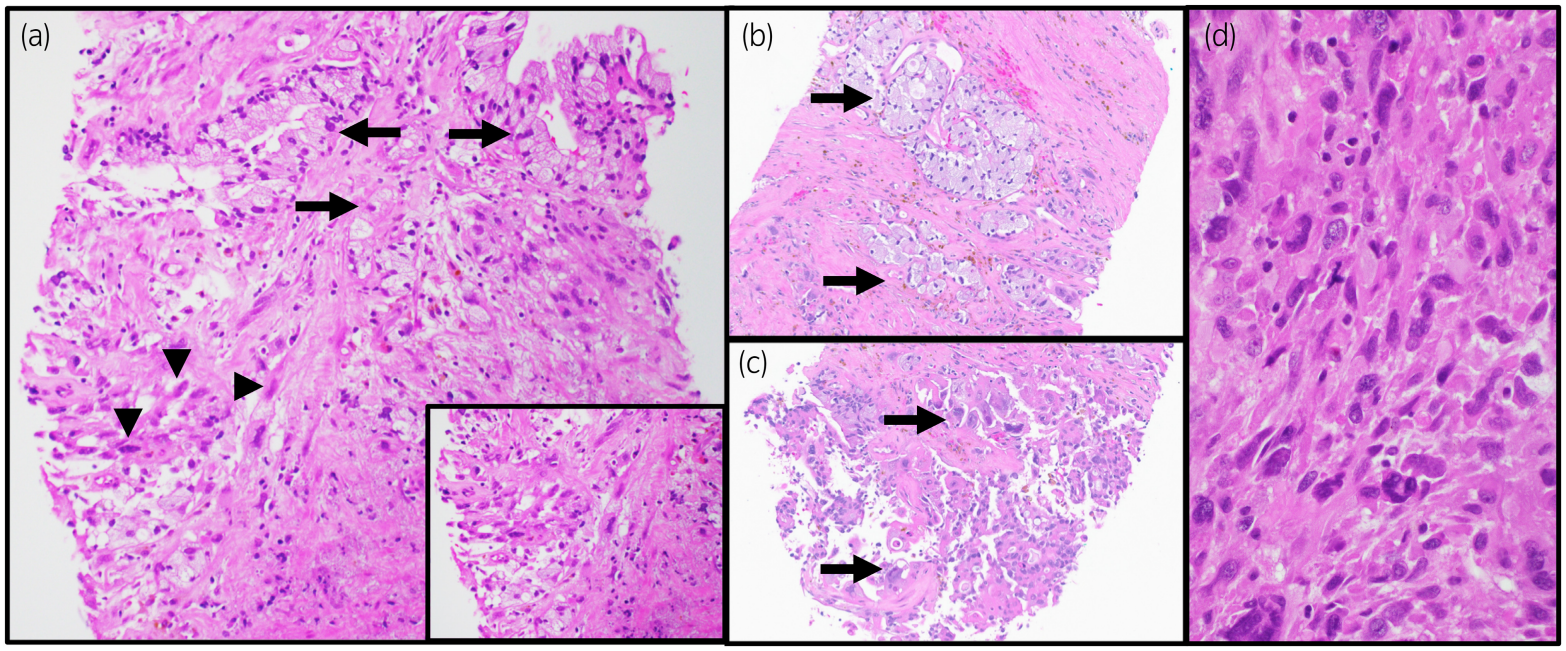
Morphology of mucin‐producing urothelial‐type adenocarcinoma of the prostate. Atypical columnar to cuboidal glandular cells containing intracytoplasmic mucin were seen infiltrating the prostate gland (a,b—arrows). The area with sarcomatoid differentiation was closely mixed with the adenocarcinoma (a, arrowheads and inset box). Pleomorphic areas of tumor cells were noted (c, arrows). Subsequent transurethral resection of the tumor demonstrating sarcomatoid areas is shown in (d). Digitally scanned H&E stained slides, 20× magnification; (a) inset box and (d), 40× magnification (Aperio GT 450, Leica Biosystems).

A transrectal ultrasound‐guided biopsy targeting six different areas of the prostate gland (bilateral apex, mid, and base) along with the designated lesion was obtained. The tumor involving all the above sites was consistent with mucin‐producing urothelial‐type adenocarcinoma of the prostate. Atypical columnar to cuboidal glandular cells containing intracytoplasmic mucin were seen infiltrating the prostate gland (Fig. 2a,b—arrows). Occasional complex glandular forms, pleomorphic single cells with hyperchromatic nuclei, and apoptotic bodies were seen (Fig. 2c, arrows). Interestingly, multifocal areas of differentiation into sarcomatoid morphology closely mixed with the adenocarcinoma were noted (Fig. 2a, arrowheads and inset box). A minor component (<5%) of conventional prostate adenocarcinoma with therapy‐related effect was seen focally, which was morphologically and immunohistochemically distinct from the dominant tumor. Notably, no urothelial carcinoma in situ or urothelial carcinoma was identified. On immunohistochemistry, the adenocarcinoma cells from the dominant tumor were positive for keratin AE1/AE3, cytokeratin 7 (CK7), carcinoembryonic antigen (CEA), and CK20 (rare positive), whereas they were negative for PSA and NKX3.1. Secondary metastases from other organs like colon, lung, and kidneys were excluded by negative staining for CDX2, SATB2, TTF‐1, and PAX8. The sarcomatoid areas were negative for all the cytokeratin immunostains. The expanded immunohistochemical staining performed is summarized in Table 1. Molecular studies performed on the tumor revealed microsatellite stability and alterations involving the following genes—*KRAS* (*Q61L* missense substitution; variant allele frequency 39.7%), *PTEN* (splice site 210‐2A > C; variant allele frequency 41.6%), *RAD21* amplification, and *TP53* (C135fs*35 deletion—frameshift; variant allele frequency 41.7%).

**Table 1:** Extended immunohistochemistry panel performed on the patient's tumor to establish a diagnosis of mucin‐producing urothelial‐type adenocarcinoma of the prostate

	AE1/AE3	CK7	CK20	CEA	NKX3.1	PSA	CDX2	SATB2	TTF‐1	PAX8	GATA3	p40	p63	CK5/14	p504S
UTA	+	+	+ (R)	+	−	−	−	−	−	−	+ (P)	+ (P)	+ (P)	RE	+
SC	−	−	−	−	−	−	−	−	−	−	+ (P)	−	−	NP	NP

NP, not performed; P, patchy; R, rare; RE, retained; SC, sarcomatoid areas; UTA, mucin‐producing urothelial‐type adenocarcinoma.

### Follow‐up

By four cycles of chemotherapy, he had radiologic improvement of tumor. At 12 months, the patient's scan redemonstrated local progression that led to a transurethral resection (TUR) of the tumor which showed predominant high‐grade sarcomatoid areas (Fig. 2d), morphologically like the previous biopsy. The patient is currently treated with pembrolizumab (anti‐PD1 antibody) with stable disease, subject to regular surveillance (radiologic and cystoscopic) and supportive care.

## Discussion

Mucin‐producing urothelial‐type adenocarcinoma of the prostate arises from the prostatic urethra and large prostatic ducts.^1^ Other entities in the differential diagnosis like primary prostatic adenocarcinoma with mucin production and secondary metastasis from primary bladder or colonic origin are more commonly encountered. Data on these cases are very limited, and ongoing effort via case studies provides an improved understanding of this entity^1^, ^2^, ^3^, ^4^, ^5^, ^6^, ^7^, ^8^, ^9^, ^10^, ^11^ (Table 2). Most patients with this tumor present with urinary obstruction, dysuria, hematuria, and mucosuria. Serum PSA values are generally less than 1.5 ng/mL,^12^, ^13^, ^14^ a sign of the tumor's origin from prostatic urethral epithelium rather than prostatic luminal cells, although some patients have shown elevated serum PSA (greater than 10 ng/mL).^4^, ^5^ Our case also showed elevated PSA with a favorable biochemical response to ADT, which is explained by co‐existing minor foci of conventional prostate adenocarcinoma demonstrating therapy‐related change, a finding that has been reported before.^8^

**Table 2:** Review of the findings from prior case studies on mucin‐producing urothelial‐type adenocarcinoma of the prostate

	Clinical presentation	Radiologic findings	Gross/Cystoscopy findings	Microscopic features and pertinent IHC	Treatment	Follow‐up
Current study	86‐year‐old, urinary obstruction, hematuria; serum PSA 4.39 ng/mL	Abscess‐like features within the prostate gland	N/A	Mucin‐producing adenocarcinoma with sarcomatoid features IHC: CK7+, CEA+, PSA−	Hormonal therapy, chemotherapy*, immunotherapy	Local progression at 12 months
Curtis *et al*. (case 1)	89‐year‐old, urinary retention, flank pain, and hematuria; serum PSA 1.17 ng/mL	N/A	Friable growth at the prostatic urethra	Mucin‐producing adenocarcinoma IHC: CK7+, CEA+, PSA−	Transurethral resection of the prostate	Metastatic disease in 9 months
Curtis *et al*. (case 2)	57‐year‐old; serum PSA 1.47 ng/mL	N/A	2.5 cm tumor bulging into the prostatic urethra	Mucin‐producing adenocarcinoma IHC: CK7+, CEA+, PSA−	Radical prostatectomy	No recurrence at 16 months
Tran *et al*. (case 1)	73‐year‐old, urinary obstruction; serum PSA <1.3 ng/mL	Enlarged prostate with heterogenous echogenicity	Cystic tumor with abundant mucus	Mucin‐producing adenocarcinoma IHC: CEA+, PSA−	Radical prostatectomy	No recurrence at 12 months
Tran *et al*. (case 2)	68‐year‐old, hematuria, serum; PSA <1 ng/mL	N/A	Lesion in the prostatic urethra	Mucin‐producing adenocarcinoma, signet‐ring cells IHC: CEA+ (focal), PSA−	Simple prostatectomy	Recurrence in 42 months
Sebasta *et al*.	81‐year‐old, irritative lower urinary tract symptoms and mucosuria; serum PSA 0.38 ng/mL	Heterogenous right prostate lobe	N/A	Mucin‐producing adenocarcinoma IHC: CK7+, PSA−	Transurethral resection of the prostate, adjuvant chemoradiation*	No progression/recurrence reported
Ortiz‐Rey *et al*.	68‐year‐old; serum PSA 11.8 ng/mL	Enlarged prostate with heterogenous right lobe	Prostatic resections with grossly mucoid appearance	Mucin‐producing adenocarcinoma, signet‐ring cells IHC: CK7+, CEA+, PSA−	Hormonal therapy, subsequent transurethral resections of the prostate	Progression in 22 months, death in 40 months
Adley *et al*.	55‐year‐old, abnormal digital rectal exam; serum PSA 10.0 ng/mL	Diffuse involvement of the left prostate peripheral zone with extension across the midline.	Firm and necrotic infiltrative mass replacing the entire left lobe, extending into the capsule and left seminal vesicle	High‐grade colonic‐type adenocarcinoma, dirty‐type necrosis, no intra or extracellular mucin IHC: CK7+ (focal), CEA+ (focal), PSA−	Radical prostatectomy	N/A
Niu *et al*.	60‐year‐old, urinary frequency and dysuria; serum PSA 3.0 ng/mL	N/A	Small amounts of mucoid material covering the prostatic urethra	Mucin‐producing adenocarcinoma, signet‐ring cells IHC: CEA+, PSA−	Transurethral resection of the prostate, radiotherapy	No progression/recurrence at 12 months
Chen *et al*.	59‐year‐old, urinary obstruction and hematospermia; serum PSA 1.0 ng/mL	Prostate lesion	Prostatic urethral tumor and an unremarkable bladder	Mucin‐producing adenocarcinoma IHC: CK7+, CEA+ (focal), PSA−	Transurethral resection of the prostate	No progression/recurrence at 12 months
Kawasaki *et al*.	66‐year‐old, urinary obstruction; serum PSA 0.186 ng/mL	Hypodense mass in the right prostate lobe	N/A	Mucin‐producing adenocarcinoma IHC: CK7+, CEA+ (focal), PSA−	Transurethral resection of the prostate, chemotherapy*	Metastatic disease in 14 months
Nova‐Camacho *et al*.	70‐year‐old, urinary obstruction; serum PSA 0.49 ng/mL	Polylobulated, hyperintense tumor with mucinous content	Ulcerated lesion covered with mucoid material in the prostatic urethra	Mucin‐producing adenocarcinoma IHC: CK7+, CEA+, PSA−	Radical cysto‐prostatectomy	Recurrence in 15 months
Peak *et al*.	80‐year‐old, recurrent hematuria status post radiation treatment** for prostate cancer; serum PSA <0.1 ng/mL	Atypical friable papillary lesion in the prostatic urethra	Large, lobulated tumor along the right prostate lobe with overlying mucous	Mucin‐producing adenocarcinoma IHC: CK7+, PSA−	Transurethral resection of the prostate	No progression/recurrence at 3 months
Shimizu *et al*.	59‐year‐old, urinary retention; PSA 0.74 ng/mL	Fluid‐retention in tumor, likely mucin	Mucin‐rich prostate tumor with possible bladder infiltration	Mucin‐producing adenocarcinoma IHC: CK7+, PSA−	Radical prostatectomy, chemotherapy*	Lung metastasis in 3 months, pelvic recurrence in 9 months.

IHC, immunohistochemistry; N/A, not available.

* Chemotherapeutic agents used in the above studies: Current study—carboplatin and paclitaxel, Sebesta *et al*.—capecitabine, Kawasaki *et al*.—gemcitabine and cisplatin, Shimizu *et al*.—capecitabine and oxaliplatin.

** One prior study (Peak *et al*.) reported that their patient was treated with radiation therapy for conventional prostate cancer and developed mucin‐producing urothelial‐type adenocarcinoma of the prostate many years later.

Histologically, the tumor shows atypical, tall, pseudostratified columnar cells (likely arising from glandular metaplasia of the prostatic urethra or proximal prostatic ducts) infiltrating the stroma. Nuclear atypia and mitotic activity have been noted along with varying degrees of necrosis, signet‐ring cells, squamous differentiation, perineural invasion, and granulomatous inflammatory response.^1^, ^4^, ^5^, ^6^, ^7^ The traditional Gleason scoring system used for prostatic adenocarcinoma does not apply to this entity as it is not derived from prostatic ducts or acini. It differs from mucinous adenocarcinoma of the prostate which often shows cords of cuboidal epithelium and cribriform glands with low‐intermediate grade cytology and tumor cells floating in pools of extracellular mucin.^9^ Differentiating non‐urachal adenocarcinoma of the bladder is a greater challenge, and involves relying on clinical, radiological, and cystoscopic examination.^2^ In our case, radiologic studies ruled out any bladder masses, and histologically we did not encounter areas of urothelial carcinoma in situ or urothelial carcinoma that may have diverged or differentiated into glands.

Our case validates the previously described morphology, immunophenotype, and reports novel molecular alterations. While most prior cases have diagnosed this entity on resection specimens, the morphologic and immunohistochemical features seen in our case were supportive of the diagnosis on a biopsy and TUR specimen. Additionally, alterations involving the *KRAS*, *PTEN*, *RAD21*, and *TP53* genes were identified. *KRAS* mutations are commonly seen in gastrointestinal, lung, and a subset of bladder adenocarcinomas,^15^ while *PTEN* alterations are seen in gynecologic tumors (breast, ovaries, and endometrium) and inherited PTEN hamartoma tumor syndromes.^16^ *RAD21* amplification is shown to correlate with poor prognosis in breast, endometrial, *KRAS*‐mutant colorectal, and hormone‐resistant prostate cancers,^17^, ^18^, ^19^ while *TP53* mutations are common in many advanced cancers.^20^ It was interesting to note that the molecular phenotype of this tumor aligned with bladder or colorectal adenocarcinomas and was comparatively distinct from reported phenotypes in conventional prostate adenocarcinoma.

Prognosis, optimal therapy, and survival data in patients diagnosed with this tumor are under‐studied. Hormonal therapy may be valuable in cases that have concomitant conventional prostate cancer; however, our patient's dominant tumor did not respond to ADT. Surgical resection, chemoradiation, and targeted therapies remain viable options. Of the identified alterations, *KRAS*, *PTEN*, and *TP53* have potential targeted therapies, and ongoing clinical trials in different tumors continue to assess the efficacy of these agents. *KRAS* mutations may predict sensitivity to MEK inhibitors, leading to better response rates in lung tumors.^21^ Tumors with *PTEN* alterations may respond to mTOR and PARP inhibitors, which have demonstrated benefits in gynecologic tumors.^22^, ^23^ Head and neck, gynecologic, and gastrointestinal tumors with *TP53* mutations have shown benefit with chemotherapy regimens combined with WEE1 inhibitor.^24^, ^25^ Lastly, immunotherapy agents like anti‐PD1 antibodies have shown promise in advanced/high‐grade urothelial tumors^26^ and are currently being explored as therapeutic options in our case. Given that ours is the first report to describe the molecular phenotype of this rare entity, the above‐targeted therapies are of potential value, however, the effectiveness remains to be known.

## Conclusions

Diagnosis, optimal therapy, and survival data in mucin‐producing urothelial‐type adenocarcinoma of the prostate are under‐studied. We contribute to the understanding of its clinical features, histologic spectrum, and unique molecular characteristics.

## Author contributions

Pranav S Renavikar: Conceptualization; data curation; investigation; writing – original draft; writing – review and editing. Thomas J Auen: Data curation; investigation; writing – original draft; writing – review and editing. David G Wagner: Writing – review and editing. Subodh M Lele: Conceptualization; supervision; writing – review and editing.

## Conflict of interest

The authors declare that they have no conflicts of interest relevant to this manuscript.

## Informed consent

Informed written consent of the patient is obtained.

## Registry and the Registration No. of the study/trial

Not applicable.

## Approval of the research protocol by an Institutional Review Board

Not applicable—the study is waived from review as determined by the University of Nebraska Medical Center Institutional Review Board.
